# Climate Change: From Science to Practice

**DOI:** 10.1007/s40572-018-0187-y

**Published:** 2018-03-05

**Authors:** Nicola Wheeler, Nick Watts

**Affiliations:** University College London, Gower St, Bloomsbury, London, WC1E 6BT UK

**Keywords:** Public health, Climate change, Barriers, BRACE, Adaptation pathways

## Abstract

**Purpose of Review:**

Climate change poses a significant threat to human health. Understanding how climate science can be translated into public health practice is an essential first step in enabling robust adaptation and improving resiliency to climate change.

**Recent Findings:**

Recent research highlights the importance of iterative approaches to public health adaptation to climate change, enabling uncertainties of health impacts and barriers to adaptation to be accounted for. There are still significant barriers to adaptation, which are context-specific and thus present unique challenges to public health practice. The implementation of flexible adaptation approaches, using frameworks targeted for public health, is key to ensuring robust adaptation to climate change in public health practice.

**Summary:**

The BRACE framework provides an excellent approach for health adaptation to climate change. Combining this with the insights provided and by the adaptation pathways approach allows for more deliberate accounting of long-term uncertainties. The mainstreaming of climate change adaptation into public health practice and planning is important in facilitating this approach and overcoming the significant barriers to effective adaptation. Yet, the immediate and future limits to adaptation provide clear justification for urgent and accelerated efforts to mitigate climate change.

## Introduction

That anthropogenic emissions of greenhouse gases (GHGs) are altering the Earth’s climate is unequivocal. Human activities have now pushed atmospheric concentrations of carbon dioxide above 400 ppm—a level not exceeded for the last 400,000 years of Earth’s history [1]. The result is a global average increase in Earth’s temperature of over 0.85 °C since 1880 [2]. Mediated through climatic systems, these rising temperatures result in stronger and more frequent extremes of weather, changes to precipitation patterns and arable land, greater ice melt, and disruptions to many other environmental processes. Humans are dependent upon the natural environment for clean air, safe drinking water, nutritious food, and shelter, with rapid changes having serious consequences for their health and wellbeing. Indeed, climate change threatens the last 50 years of gains made in public health [3•, 4].

Climate change affects human health through three pathways. The direct health impacts of climate change are associated with the changing frequency and severity of extreme weather events, such as heat, drought, and heavy rain. Secondly, climate change’s effects can be mediated through natural systems, for instance, by altering the burden and pattern of distribution of vector-, water-, and food-borne diseases. Finally, climate change may impact health indirectly, via social institutions, resulting in undernutrition (for example, when climatic factors interact with global food markets), mental ill-health, and even violence and conflict [3•]. Whilst it is widely recognised that climate change affects human health, attributing morbidity and mortality to climate change is challenging, for the attribution of individual weather events to climate change itself is difficult, even without accounting for the associated human health consequences and mediation through ecosystems and human institutions. Nonetheless, the ability to attribute events to climate change is improving, and consequently, there is growing evidence that climate change is already impacting human health. For instance, the European heatwave of 2003 killed over 70,000 people, with France being particularly badly affected; climate change exacerbated the severity of this heatwave and is estimated to have increased the risk of heat-related mortality in Paris by 70% [5, 6]. Such research on the health impacts of climate change is vital to effectively addressing climate change; in particular, understanding health impacts is important in enabling the public health sector to adapt to climate change and reduce associated health burdens. Adapting to climate change is an immense challenge for public health practice, owing to the unpredictability of its impacts on health; the rapidity of changes in the climate system relative to historical stability; and that it simultaneously impacts multiple risk factors and determinants of health, thus correlating previously uncorrelated risks. Understanding how climate science can be translated into public health practice is therefore an essential first step in enabling robust adaptation to climate change.

Adaptation is “the process of adjustment to actual or expected climate and its effects”. In human systems, adaptation is intended to reduce or avoid risks [7]. Health adaptation to climate change can be categorised into three forms. Incidental adaptation is associated with actions taken within the health sector that aid climate change adaptation, but are not delivered for this express purpose. Linear adaptation activities are responses to specific climate threats through the implementation of adaptive practices that enable health systems to respond to identified risks. Finally, building resilience is about system-wide changes that improve the ability of a system and society to cope with climate change.

This paper reviews the recent literature on health adaptation, considering the ways in which public health practice can best make use of climate science to inform robust adaptation. How the essential services of public health relate and can be transferred to climate change adaptation will then be discussed. Barriers to the public health sector adapting to climate change will subsequently be outlined and means of overcoming these barriers discussed through some key frameworks on public health adaptation to climate change and mainstreaming climate change into public health practice. Finally, the technological, financial, and physiological limits to adaptation will be highlighted and the implications they have on an effective response to climate change. It will be argued that combining an ‘adaptation pathways approach’ with the BRACE framework (Building Resilience Against Climate Effects) is important in order to integrate long-term uncertainties into short-term adaptation practices. Secondly, that mainstreaming climate change into existing public health practice is the most effective way for public health practice to adapt to climate change and overcome some barriers to adaptation. Finally, this review will conclude with a brief note on the limits to adaptation—noting the importance of a robust, complementary mitigation strategy in order to prevent the most severe health impacts of climate change.

## Applicability of Essential Public Health Services to Climate Change

Given the degree of committed warming locked-in by historic GHG emissions, some degree of adaptation to climate change is essential [8]. Whilst climate change is a global phenomenon, its effects are felt locally, and hence, effective health adaptation strategies are largely context-specific, with the extent and nature of adaptation required of health systems dependent upon the current health status of a given population. Indeed, this is often one of the most important factors in determining the health impacts of, and responses to, climate change [9]. Whilst socioeconomic and geographical factors are clearly important as well, much of health adaptation planning has thus-far focused on strengthening existing health system functions [10, 11, 12•]. In delivering this, public health practice may be ideally placed, with its heavy reliance on data and evidence to inform actions; its familiarity with the need to engage multiple sectors and processes within and beyond the formal healthcare system; and its preparedness for responding to unexpected and non-linear events. Indeed, many of the health impacts of climate change represent challenges and threats the health system is already facing in one way or another. To the extent that health adaptation to climate change can be aligned with efforts to strengthen health systems more generally, such interventions represent “no-regrets” options, to be targeted and maximised [13].

Despite these synergies, climate change demands two primary responses from public health practice: the improvement and strengthening of existing core functions to tackle amplified, but previously experienced, risks; and the ability to respond to new and unseen health threats (at least in that given geography), demanding novel management strategies [12•, 14, 15].

Existing classifications of public health services—such as the distinction of primary, secondary, and tertiary prevention—can be helpfully applied to climate change adaptation, and can in turn, be used as guidelines for integrating adaptation into public health practice. Primary prevention is associated with the prevention of disease onset. Secondary prevention works to reduce the body-wide impacts diseases can have, through treatment to prevent further and more severe risks in future. Tertiary prevention aims to reduce the social and physical impacts of diseases and their symptoms, to best ensure people can maintain a good quality of life. These three principles directly relate to climate change responses. Primary prevention involves reducing population exposures to climate change; secondary prevention is concerned with the avoidance of adverse health outcomes given inevitable exposure; and tertiary prevention decreases long-term, and more systemic health impacts of climate change [8]. In much the same way as prevention can be split into three tiers, so too can the effects of climate change on health be categorised as primary, secondary, and tertiary impacts [16]. These are complementary to the three prevention categories outlined above and can be used to help inform effective adaptation.

Beyond prevention, public health activities can be broadly categorised into three functions: assessment (collecting, analysing and disseminating data), policy development (for policies within and relevant to the health sector), and assurance (connecting people with services). All of these can be applied to health adaptation to climate change [12•]. Table 1 summarises ten essential public health services (as described by the US Department of Health and Human Services) within these three categories, and provides examples of how these might be translated for the purposes of climate change adaptation [12•, 17].

**Table 1:** Essential public health services and their practical translation into climate change adaptation within public health practice

Public health service	Translation to climate change adaptation
1. Monitor health status to identify community health problems	Surveillance for environmental risks, vulnerability and disease
2. Diagnose and investigate health problems and health hazards in the community	Vulnerability assessments and health impact assessments
3. Inform, educate and empower people about health issues	Public health communication about climate change
4. Mobilise community partnerships to identify and solve health problems	Interdisciplinary and inter-sectoral collaboration among stakeholders
5. Develop policies and plans that support individual and community health efforts	Climate-related event preparedness planning and mitigation policies maximising health co-benefits
6. Enforce laws and regulations that protect health and ensure safety	Legislation to require enhanced building code and regulations to respond to extreme weather events
7. Link people to needed personal health services	Personalised early-warning systems for patients with climate-sensitive health problems
8. Ensure presence of a competent public health and personal healthcare workforce	Training of health professionals about climate change and its health impacts
9. Evaluate effectiveness, accessibility and quality of personal and population-based health services	Evaluate preparedness plans, health communications strategies and other initiatives
10. Conduct research to attain new insights and innovative solutions to health problems	Research to provide data-based support for public health action on climate change

(Sources: Adapted from Frumkin et al. 2015 [12•], by permission of Oxford University Press, USA; and adapted from Frumkin et al. 2008 [17])

Whilst these principles provide an excellent basis to build upon, health adaptation must carry out these functions with relative uncertainty about the future [12•]. Public health already manages the projection of future health risks, such as disease outbreaks; however, these risks occur within relatively predictable and often previously observed boundaries. This added uncertainty necessitates the need for public health practitioners to engage with new expertise—such as climatologists—to better understand the future risks and to inform planned responses.

Building upon the ten essential public health services presented above (and their translation to climate change adaptation), it is important to consider what principles characterise an effective approach. Frumkin, Hess, and Luber argue that there are seven key characteristics of the ideal public health response to climate change [12•]. Firstly, and perhaps most demandingly, it must have its foundations in a robust public health system, able to deliver core health services across its whole population. Secondly, any response requires clear understanding of the nature of the environmental hazards, to ensure they are robust and effectively and efficiently reduce health risks associated with climate change. Thirdly, “all-hazard preparedness” should be prioritised, ensuring a system is able to cope with all climate hazards. Fourthly, health systems should emphasise resilience, stressing the need for building strong communities and systems with the ability to cope with unpredictable stressors. Fifthly, any potential co-benefits in addressing climate change should be maximised; for example, ecosystem-based adaptation can have mental health benefits in exposing people to ecosystem services. Sixthly, strong and cross-sectoral partnerships are essential, both for climate data provision and interpretation for the health sector, but also because climate change will impact a range of sectors and risk factors, all of which must respond in order safeguard public health. Lastly, adaptive management, which focuses on institutional learning and constant evaluation, should be promoted [12•].

## Barriers to Public Health Practice Adapting to Climate Change

Few health systems have the financial or organisational capacity to undertake whole-sale adaptation, incorporating all of the interventions described above [12•]. Indeed, health systems in low- and middle-income countries are often overwhelmed with the day-to-day delivery of basic health services. These interact with a range of other challenges to present significant barriers to effective health adaptation. Broadly defined in this context, barriers are factors that “make it harder to plan and implement adaptation actions or that restrict options” available [18]. Defining barriers to adaptation is complex, as many are highly context-specific and dependent upon the sectors, geographies, and time scales involved [19]. That being said, barriers generally fall within four broad categories: economic, informational, institutional or political, and social [18–21]. Economic barriers are those associated with limited or absent financial resources to invest in the research and interventions required for adaptation. Whilst financial constraints are the most commonly referenced barrier to adaptation, and that which may prevent the initiation of an adaptation intervention, they are clearly not the only important barrier. Adequate resourcing is a necessary, but insufficient condition for effective adaptation. Informational barriers make long-term planning difficult, with climate modelling and interpretation of the corresponding health outcomes, often resource intensive and rarely available in a timely manner or at the spatial resolution required. Institutional and political barriers limit organisations’ and governments’ abilities to understand and respond to system-wide changes, with change management and management under significant uncertainty often particularly challenging. Finally, these issues are often compounded by highly complex social barriers, arising as a result of specific values and cultures both within organisations and affected populations.

Financial, informational, institutional, and social barriers can be identified in many of the adaptation responses to climate change. These often interact with other societal challenges in low- and middle-income countries, ranging from high levels of poverty and hence of baseline vulnerability, weak social institutions, and the perception of more pressing issues to be managed. However, three climate change-specific barriers are repeatedly referenced in the literature [19].

Firstly, reconciling the relatively long-term and distant impacts of climate change (perceived or otherwise) with the short-term dynamics of politics and decision-making is often highly problematic. Across the world, demographic factors and technological changes are placing health systems under extraordinary pressure, consuming resources, and demanding the attention of policymakers at the expense of long-term adaptation planning, which is more easily put-off. Secondly, a reliance on climate models to identify and inform problems and solutions presents novel challenges to public health practice, introducing new and complex concepts to a profession that is largely inexperienced and untrained in climate change. Thirdly, and perhaps least-surprisingly, large uncertainties surrounding the health impacts of climate change (and indeed, what degree of climate change to account for) render traditional, often linear, planning techniques less effective [19].

## Overcoming Barriers to Adaptation in Public Health Practice

Whilst a number of the barriers faced by countries and communities may be planned for and successfully avoided ahead of time, this is not always the case. Given the highly context-specific nature of many of these barriers, they may not always be identifiable a priori, but instead reveal themselves during the implementation phase of an intervention (after initial financial resourcing barriers may have been overcome) [20]. The often complex and uncertain nature of the links between climate change and public health mean that linear approaches to adaptation may be more likely to be insufficient.

Here, a flexible approach to adaptation management is needed, with more iterative models that not only account for unforeseen problems, but embrace them. Implementing iterative models and reforming practices can be difficult. This is seen regularly in rapidly emerging economies, where multiple sectors are changing simultaneously. Often, capacity constraints result in institutions simply altering their structure and appearance rather than their actions and functions, resulting in “capability traps” which cause systems to stagnate or deteriorate. To avoid these traps, Andrews et al. propose a Problem-Driven Iterative Adaptation (PDIA) approach to complex social interventions. PDIA is built upon four principles: it focuses on solving locally selected and defined problems; it encourages deviation and experimentation from authorised decision-making; it embeds this experimentation into feedback loops to facilitate quick learning; and it deliberately engages a wide range of stakeholders to promote viable, legitimate, relevant, and supportable reforms [22]. Such models for iterative learning are well-established in the broader development literature, simply requiring application to public health adaptation to climate change.

PDIA and concepts of adaptive management—focusing on continued learning, extensive stakeholder input, and a Bayesian approach to evidence and decision-making—are concepts familiar to much of public health practice [14, 15]. For adaptive management to be effective, it must regularly assess management objectives; fully understand the needs of the system adaptation is occurring within; implement a range of management decisions; monitor and evaluate their outcomes; be willing to adapt its approach based on this evaluation, incorporating learning into future actions; and offer a collaborative structure for stakeholder participation and learning [23]. Undertaking such an approach in the context of climate change requires specific tools, notably vulnerability assessments (to identify the most at-risk populations), climate modelling (to project health impacts), and decision-support mechanisms (to avoid maladaptation).

Whilst the principles of PDIA and adaptive management may be familiar to public health practice, there is still some challenge in applying them to a novel issue such as health adaptation to climate change [24•]. Indeed, for most public health practitioners, translating climate science into public health practice requires additional capacity and planning [25]. Knowledge of climate change is incomplete in public health practice, and there is generally inadequate staffing and training to improve this understanding, leaving public health systems feeling unprepared and unable to cope. Thus, frameworks specifically outlining how public health systems should adapt to climate change are invaluable. Here, the five steps of the BRACE Framework (“building resilience against climate effects”)—developed by the Centers for Disease Control and Prevention (CDC)—provide a widely cited and implemented example of an iterative approach:

1. Anticipating climate impacts and assessing vulnerabilities
2. Projecting the disease burden
3. Assessing public health interventions
4. Developing and implementing a climate and health adaptation plan
5. Evaluating impacts and improving the quality of activities

The process encourages public health systems to use the best available climate science to project health impacts and prioritise adaptation interventions [26]. By reinforcing a well-established commitment among health practitioners of evidence-based practice, the BRACE framework also increases engagement within the sector. Moreover, the framework recognises that complex systems (such as climatic and social systems) may not be fully understandable, interventions may have unintended outcomes, and management strategies need to be updated regularly through iterative, learning-based approaches [24•].

Step one of the BRACE framework is aimed at improving understanding of future climate change, probable health effects, and populations and systems which are most vulnerable; this is obtained through climate data and vulnerability assessments to understand exposures and sensitivities. The second step models the burden of a range of climate-sensitive diseases and is designed to be updated regularly with new information fed into adaptive management processes. Step three requires assessing and identifying the most appropriate adaptation options, both in terms of identifying adaptation responses to specific health risks, and by developing strategies to improve baseline community resilience. The fourth step incorporates the first three steps of the framework to develop a comprehensive health and adaptation plan; this should complement any existing climate change plans and incorporate best practices. The final stage encompasses the iterative element of the framework, evaluating interventions and identifying barriers to effective adaptation, feeding back into the relevant stage [24•]. The BRACE framework provides a powerful means of supporting health system adaptation to climate change and is already being trialled in 16 states and two cities in the USA. For instance, MN has developed an Extreme Heat Toolkit; San Francisco is developing indicators to assess community resilience to climate change; ME is implementing improved monitoring of vector-borne diseases; and MI has increased state and local level capacity to undertake health impact assessments that incorporate climate change [25].

**Fig. 1:**
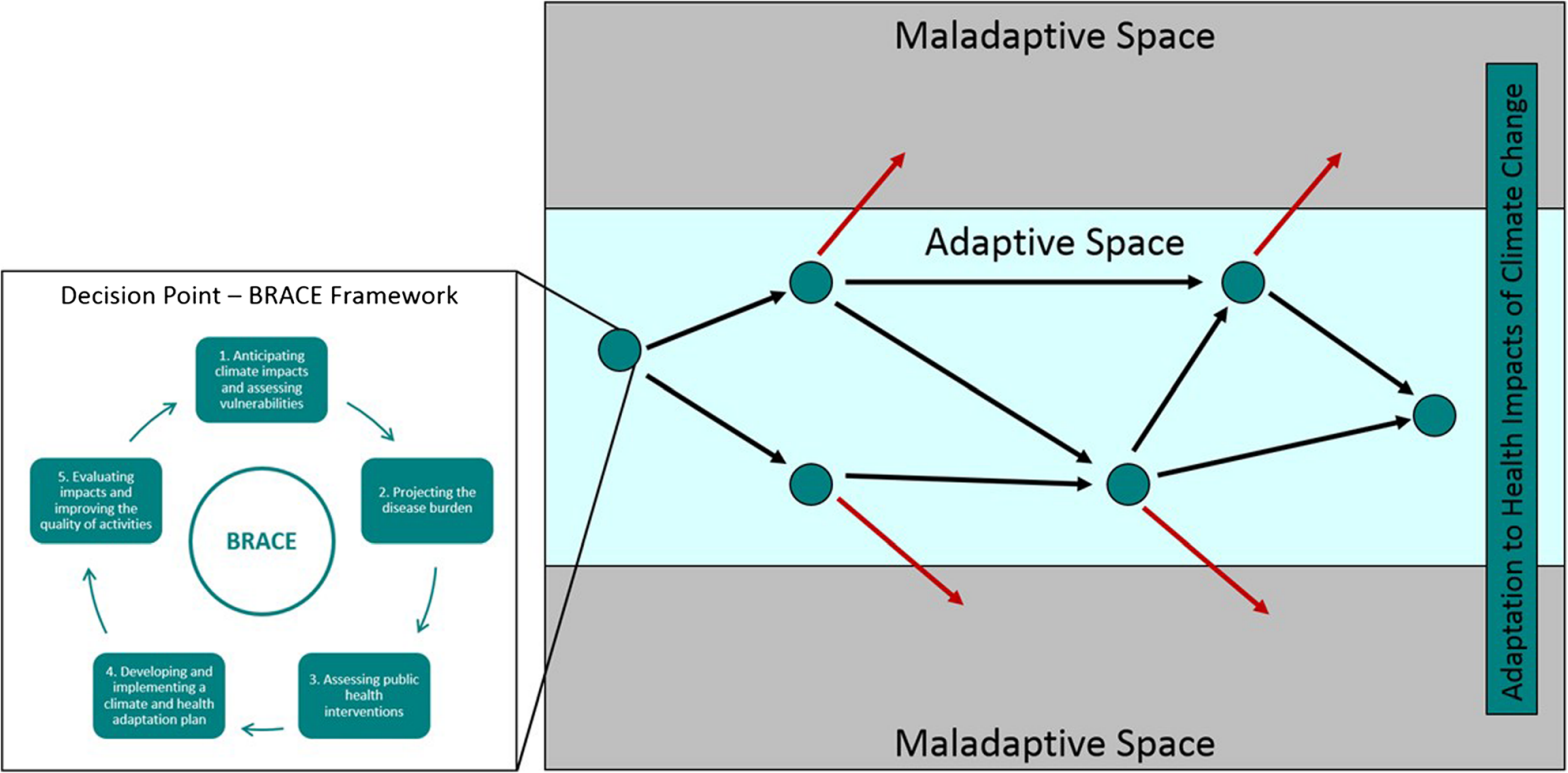
Adaptation pathways approach incorporating the BRACE framework at each decision point along the pathways to adaptation to the health impacts of climate change. The *circular points* represent the decisions points where the next stages of adaptation are implemented. The *black arrows* show different adaptation options that promote robust adaptation and *red arrows* represent maladaptive choices. The end point is successful adaptation to the health impacts of climate change, which can be achieved by a range of pathways and is informed by the implementation of the BRACE framework at each decision point [24•, 27•]. (Sources: Adapted from Marinucci et al. [24•]; and Reprinted from Wise et al. [27•], with permission from Elsevier)

Complementing the BRACE framework, an ‘adaptation pathways approach’ has been designed to explicitly function over longer timescales and scenarios, incorporating the long-term evolution of climate change into short-term adaptation decisions, and so promoting robust decision-making under uncertainty (Fig. 1, right panel) [28]. An adaptation pathways approach establishes a strategic vision for the future and plans for a wide variety of possible climatic and social scenarios over an extended time horizon, establishing adaptation responses to each of these combinations at successive moments in the future. During this process, decision points are identified, at which point choices are made regarding the required adaptation under a certain climate outcome.

For example, the Environment Agency in England has adopted an adaptation pathways approach in planning the defence of London and the Thames Estuary against sea level rise. Future climate change scenarios and population projections were modelled, and the extent of sea level rise and populations exposed associated with each scenario were estimated. Adaptation plans and interventions were then developed for the full range of future climatic and social scenarios, with various decision trigger-points identified (for example, adaptation action to be taken with 0.5 m of sea level rise with a more exposed population), ensuring that the right decisions were made at the right time and financial resource was invested most effectively [29]. Ultimately, an adaptation pathways approach provides a route map reaching a common, ideal end point (in this case, in-tact flood defences for London). An adaptation pathways approach also helps to avoid maladaptive choices, as it demands regular evaluation and establishes strategic decision points designed to trigger the implementation of the most effective adaptation action given a specific scenario. However, it also gives a suite of options leading to this end point (see Fig. 1). This enables flexibility in short-term decisions, allowing appropriate adaptation actions to be adopted according to how climate change evolves over time. Hence, robust, flexible and no-regret adaptation actions are promoted from the start [28].

Here, it is proposed that these two models might function best together, with the BRACE framework conceptualised as an individual decision point of each adaptation pathways approach, encouraging long-term health adaptation planning, through a flexible pathways approach designed specifically for climate change adaptation within public health practice (Fig. 1) [24, 27•].

## Mainstreaming Climate Change Into Public Health Practice

Mainstreaming climate change adaptation as a core function of public health planning provides an effective method for overcoming many of the preliminary barriers described above [30]. Whilst any such mainstreaming will present significant challenges, the cost-savings and social benefits that result should far exceed the upfront costs. Funding to improve the ability of existing public health services to adapt to climate change may be found in a variety of public and private sources. In the context of low- and middle-income countries, five funding mechanisms for climate change adaptation are notable: the World Bank’s Pilot Program for Climate Resilience, the European Commission’s Global Climate Change Alliance of the European Union, the United Nations Framework Convention on Climate Change’s (UNFCCC) Adaptation Fund, the UNFCCC’s Global Environmental Fund, and the UNFCCC’s Green Climate Fund [13, 31]. In the context of declining international funding for global public health, such resources may be vital in supporting core health system strengthening and improving public health adaptation to climate change.

National Adaptation Programmes of Action (NAPAs) are climate change adaptation plans undertaken by Least Developed Countries under the UNFCCC, providing vital information on vulnerability and adaptation challenges. The NAPAs are thus a key source of information in determining the distribution of funding, which is allocated principally based on assessed vulnerability. However, as the quality of NAPAs is variable and the extent to which NAPAs incorporate health is often limited, public health outcomes are not necessarily fully accounted for when funding decisions are made; for instance, only one in four NAPAs include a comprehensive health vulnerability assessment, despite this being a key stipulation under the UNFCCC [13, 32]. National Adaptation Plans (NAPs) build upon NAPAs to identify priority actions to respond to urgent adaptation requirements. The NAP process aims to support medium- and long-term adaptation planning for all countries, and acts as a vital means of identifying funding needs and adaptation priorities [33]. Whilst historical engagement from the health sector in the development of the NAPs has been minimal, the World Health Organization (WHO) is increasingly successful in supporting countries’ (most especially low- and middle-income countries) to develop robust Health National Adaptation Plans (HNAPs). Improved public health participation in these processes should work to increase their access to adaptation funding (which has so far been concerningly low, compared with other sectors) and so contribute to mainstreaming adaptation throughout the health sector [34].

Such engagement from the health sector should be met with increased recognition from the main climate change funding bodies, which do not currently focus on health nor stipulate proportions of funding for health [13]. Commitments to these funding mechanisms have increased in recent years; for example, the Green Climate Fund is aiming to mobilise USD 100 billion by 2050 and already has USD 10.3 billion at its disposal [35, 36]. Such progress presents huge opportunities for public health practice to obtain funding for climate change adaptation and to mainstream such actions into existing health practices.

These high level considerations clearly form vital components to mainstreaming climate change adaptation into public health practice, and indeed provide the enabling mechanisms to adapt, but ultimately, it is on-the-ground actions that show the true extent to which mainstreaming is effective and thus help build the evidence base of why such plans and funding are needed. One area where significant progress has been made in mainstreaming climate change adaptation into public health practice is the provision of effective forecasting of health-relevant weather and climate risks; this is a key element of the BRACE framework and hence integral to health adaptation. Such health-tailored climate services are not only being increasingly recognised for their importance, but they are also being deployed evermore through partnerships between meteorological and health organisations. These climate services are helping public health practitioners to improve the ability of health services to detect, monitor, predict, and manage health risks associated with climatic changes. There are important tangible benefits to using these services, including reduced deaths and numbers affected by climate and weather-related events; more efficient and effective use of resources; and decreased strain on health delivery systems, via better planning and increased awareness and preparedness of climate and weather health risks. While potentially hugely beneficial, the deployment of climate services for public health practice is complex and context-specific. Some key considerations, which should help to promote the most effective use of climate services in public health practice, include: creating an enabling environment between health and meteorological services; building the capacity of these services; researching how climate services are most effective for public health in a given context; developing appropriately tailored climate services; applying these services into real public health practice; and finally evaluating the effectiveness of the climate services used to promote learning and improvement [26].

## Limits to Adaptation

There is an important distinction between barriers to adaptation and limits to adaptation. In general, barriers are possible to overcome, whereas limits are absolute and unsurmountable [20]. Having discussed the barriers to public health adaptation to climate change, it is important to briefly acknowledge the limits to adaptation. Largely, projections of the health burden of climate change are based on assumptions of a global average 2 °C temperature rise; yet it is increasingly unlikely that this target will be achieved [3•]. Therefore, understanding the technological, financial, and physiological limits of health adaptation are important in revealing the degree of climate change mitigation required. For example, these may take the form of technological limits to the size and strength of future sea walls and flood defences; or to the financial limits associated with the cost of providing safe drinking water in water-stressed regions of the world. A range of limits to human physiology exist as well. Core body temperatures reach fatal levels under sustained periods of wet-bulb temperatures over 35 °C [37]. By 2071, there is estimated to be 151,500 heat-related deaths in Europe every year, representing a 5400% increase from the average of 2700 annual deaths between 1981 and 2010, and equating to 99% of total future weather-related deaths in Europe [38]. Furthermore, already 30% of the world’s population is annually exposed to heat events that could result in deaths among vulnerable populations; this is expected to increase to 48% under a low emissions scenario, and 74% under a high emissions scenario [39].

These, and many other significant limits to adaptation provide clear justification for urgent and accelerated reductions in GHG emissions, with adaptation alone unable to protect public health from climate change.

## Conclusion

This paper has provided an overview of the ways in which climate change and climate science might best be integrated into existing public health practice, to strengthen and inform health adaptation. Climate change presents a significant challenge for the health sector and public health practice. However, the skills needed to adapt are already deployed in public health—they just need to be translated to climate change adaptation. Here, a modified version of the BRACE framework has been proposed to help promote more effective adaptation. By incorporating an adaptation pathways approach into the BRACE framework, this could help account for the long-term uncertainties of climate change and overcome some of the barriers to climate change. Mainstreaming climate change into public health practice should also streamline adaptation and help to overcome some of the barriers to adaptation planning and implementation getting underway; the benefits of this is already demonstrated by the increasing deployment of climate services in public health practice. However, there are clear limits to adaptation. Mitigation of climate change is therefore absolutely essential, for adaptation alone will not protect human health against all of the impacts of climate change.
